# The Geraniin-Rich Extract from Reunion Island Endemic Medicinal Plant *Phyllanthus phillyreifolius* Inhibits Zika and Dengue Virus Infection at Non-Toxic Effect Doses in Zebrafish

**DOI:** 10.3390/molecules25102316

**Published:** 2020-05-15

**Authors:** Juliano G. Haddad, Dovilė Grauzdytė, Andrea Cristine Koishi, Wildriss Viranaicken, Petras Rimantas Venskutonis, Claudia Nunes Duarte dos Santos, Philippe Desprès, Nicolas Diotel, Chaker El Kalamouni

**Affiliations:** 1 Université de la Réunion, INSERM U1187, CNRS UMR 9192, IRD UMR 249, Unité Mixte Processus Infectieux en Milieu Insulaire Tropical, Plateforme Technologique CYROI, 94791 Sainte Clotilde, France; juliano.haddad@univ-reunion.fr (J.G.H.); wildriss.viranaicken@univ-reunion.fr (W.V.); philippe.despres@univ-reunion.fr (P.D.); 2 Department of Food Science and Technology, Kaunas University of Technology, Radvilėnų pl. 19, Kaunas LT-50254, Lithuania; dovile.grauzdyte@ktu.lt (D.G.); rimas.venskutonis@ktu.lt (P.R.V.); 3 Laboratorio de Virologia Molecular, Instituto Carlos Chagas, ICC/FIOCRUZ/PR, Curitiba 81350-010, Brazil; ackoishi@gmail.com (A.C.K.); clsantos@fiocruz.br (C.N.D.d.S.); 4 Université de La Réunion, INSERM, UMR 1188 Diabète athérothrombose Thérapies Réunion Océan Indien (DéTROI), 97490 Saint-Denis de La Réunion, France; nicolas.diotel@univ-reunion.fr

**Keywords:** zika virus, dengue virus, medicinal plants, antiviral activity, geraniin, *Phyllanthus phillyreifolius*, zebrafish

## Abstract

The mosquito-borne viruses dengue (DENV) and Zika (ZIKV) viruses are two medically important pathogens in tropical and subtropical regions of the world. There is an urgent need of therapeutics against DENV and ZIKV, and medicinal plants are considered as a promising source of antiviral bioactive metabolites. In the present study, we evaluated the ability of *Phyllanthus phillyreifolius*, an endemic medicinal plant from Reunion Island, to prevent DENV and ZIKV infection in human cells. At non-cytotoxic concentration *in vitro*, incubation of infected A549 cells with a *P. phillyreifolius* extract or its major active phytochemical geraniin resulted in a dramatic reduction of virus progeny production for ZIKV as well as four serotypes of DENV. Virological assays showed that *P. phillyreifolius* extract-mediated virus inhibition relates to a blockade in internalization of virus particles into the host cell. Infectivity studies on ZIKV showed that both *P. phillyreifolius* and geraniin cause a loss of infectivity of the viral particles. Using a zebrafish model, we demonstrated that administration of *P. phillyreifolius* and geraniin has no effect on zebrafish locomotor activity while no morbidity nor mortality was observed up to 5 days post-inoculation. Thus, *P. phillyreifolius* could act as an important source of plant metabolite geraniin which is a promising antiviral compound in the fight against DENV and ZIKV.

## 1. Introduction

Mosquito-borne dengue (DENV) and Zika (ZIKV) viruses belonging to the flavivirus genus (Flaviviridae family) are two major concerns in public health worldwide [1]. The geographic range of DENV spreads from tropical to subtropical regions [2,3]. Most hyperendemic regions are located in Southeast Asia and Latin America where severe outbreaks of dengue disease occur annually. The four serotypes of DENV (DENV-1 to DENV-4) can cause clinical symptoms ranging from dengue fever to a severe form of disease with hemorrhage causing severe shock impairing patient vital prognosis [2]. No antiviral therapy is available for DENV infection, and prophylatic strategies are needed to prevent severe forms of dengue disease. ZIKV has become a major public health issue in the last decade [4,5]. ZIKV can be divided into African and Asian lineages [6]. Emergence of Asiatic strains of ZIKV in the South Pacific and then in the Americas in 2015 has been associated with severe neurological complications in humans [7,8,9,10]. ZIKV infection in humans can cause severe neurological defects such as congenital Zika syndrome (CZS) and Guillain–Barré syndrome [11,12]. Sexual contact, blood transfusion, and intrauterine transmission have been documented as non-conventional transmission routes for ZIKV [4,8,13]. Moreover, ZIKV can be detected in human biological fluids for prolonged periods after the infection [14,15,16]. Such findings highlight the need for effective disease prevention measures, especially with regards to the risk of CZS and the presence of viral reservoirs in human fluids such as semen [17,18].

Flaviviruses have a positive single-stranded RNA (ssRNA) of approximately 11 kb [19]. Genomic RNA is initially translated into a large polyprotein, which is cleaved co- and post-translationally, by cellular and viral proteases, into three structural proteins (C, prM, E) responsible for the morphology of the viral particle and seven non-structural proteins (NS1, NS2A, NS2B, NS3, NS4A, NS4B, and NS5) implicated in the viral replication step and virus subversion to innate immune responses [19]. ZIKV binds to cellular receptors by its E protein [20,21]. Virus particles are internalized via a clathrin-mediated endocytosis, and the fusion between the viral and endosomal membranes leads to the decapsidation of the virus and the release of the viral RNA into the cytoplasm [19]. The viral genomic RNA is then replicated and translated into viral proteins in the vicinity of the endoplasmic reticulum membranes where virus assembly can occur. The newly assembled virus particles are transported in the secretory pathway and then released into the extracellular compartment where they become fully infectious [19].

The World Health Organization (WHO) estimated that about 80% of population use traditional medicine in order to fight pathogens [22]. Indeed, several *in vitro* and in vivo studies showed that plant-derived products represent therapeutic agents against different medically important viruses, giving a hopeful view about a future naturally derived antiviral agent. Phytochemicals including polyphenols, flavonoids, alkaloids, and curcuminoids have been reported to inhibit flavivirus infection [23,24,25,26,27,28,29,30]. We recently showed that *Phyllanthus phillyreifolius*, an endemic medicinal plant from Reunion island, used locally to treat diarrhea, fever, venereal diseases, and pain due to kidney stones [31], exerts a strong antioxidant activity due to its richness in polyphenols, geraniin in particular [32,33]. Furthermore, geraniin has been demonstrated to display antimicrobial, antioxidant, anticancer, analgesic, antiviral, and immune-modulatory properties and therapeutic effects on cardiovascular diseases [32,34,35,36,37,38]. The purpose of our study was to determine whether *P. phillyreifolius* extract exerts antiviral effect against DENV and ZIKV *in vitro*. We showed that *P. phillyreifolius* extract inhibits virus infection *in vitro* and does not exhibit acute toxicity in vivo in zebrafish. We propose that antiviral action of *P. phillyreifolius* extract relates to its major compound geraniin which acts as a potent flavivirus inhibitor at the doses displaying no acute toxicity effects on zebrafish and without modifying its locomotor activity that could constitute a sign of stress.

## 2. Results

### 2.1. ZIKV and DENV Are Inhibited by an Ethanolic Extract of Phyllanthus phillyreifolius

**Figure 1:**
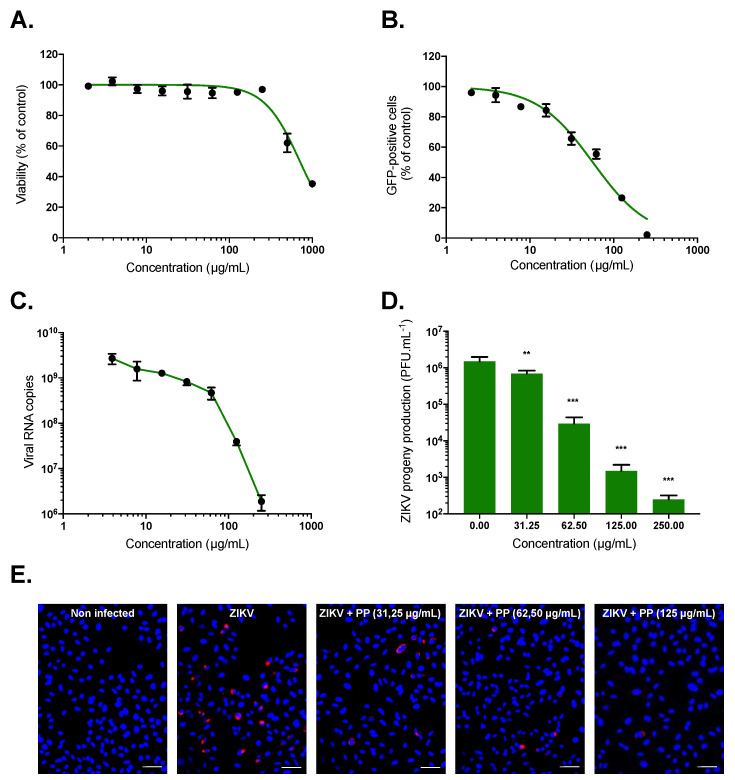
*Phyllanthus phillyreifolius* extract exhibits antiviral activity against African and Asian strains of Zika virus (ZIKV). (**A**) A549 cells were incubated with two-fold serial dilutions (1000 to 2 µg/mL) of plant extract for 72 h. Cell viability was evaluated using an MTT assay. (**B**) A549 cells were infected with ZIKV^GFP^ at MOI of 1 in presence of different concentrations (250, 125, 62.5, 31.25, 15.60, 7.80, 3.90, and 2 µg/mL) of *P. phillyreifolius*. Flow cytometric analysis of GFP fluorescence was performed 24 h post-infection. A549 cells were infected with ZIKV-PF13 at MOI of 2 and continuously incubated with increasing concentrations of *P. phillyreifolius* (PP). (**C**) The amount of viral genomic RNA in ZIKV-PF13-infected A549 cells was determined by RT-qPCR. (**D**) ZIKV progeny production was quantified by plaque-forming assay. The results shown are means ± SD of four independent experiments and are expressed as relative value compared to untreated infected cells. ** *p* < 0.01; *** *p* < 0.001, n.s. = not significant. (**E**) Immunofluorescence analysis of viral protein expression in ZIKV-PF13-infected A549 cells. The ZIKV (red) and nuclei (blue) were visualized by fluorescence microscopy. Scale bars are 50 μm. Results from a representative experiment (n = 3 repeats) are shown.

Prior to evaluating the antiviral activity of *P. phillyreifolius* extract against ZIKV, we determined its maximal non-cytotoxic concentrations (MNTC) on human epithelial cell lines A549 using an MTT assay, which assesses cell metabolic activity (Figure 1A). Plotting cell viability against different concentrations of *P. phillyreifolius* extract revealed concentration-dependent toxicity in A549 cells (Figure 1A). The concentration that inhibits 50% (CC_50_) of cell viability was calculated as 715 µg/mL (Table 1). A concentration of 250 µg/mL of *P. phillyreifolius* extracts that maintained 95%–100% of cell viability (Figure 1A) was consequently chosen for testing potential anti-ZIKV activity. A chimeric molecular clone of African strain of ZIKV expressing a GFP reporter gene (ZIKV^GFP^) was used for monitoring viral infection by flow cytometer in A549 cells where ZIKV can replicate efficiently [39]. Thus, A549 cells were infected 24 h with ZIKV^GFP^ in the presence of different non-toxic concentrations of *P. phillyreifolius*. A dose-dependent effect of *P. phillyreifolius* on ZIKV infectivity was observed with a complete inhibition of ZIKV infection at non-cytotoxic concentrations of plant extract (Figure 1B). The concentration that inhibits 50% of ZIKV infection (IC_50_) was calculated as 55 µg/mL for *P. phillyreifolius* (Table 1).

To further validate the anti-ZIKV effect of *P. phillyreifolius*, A549 cells were infected with the epidemic Asian strain ZIKV-PF13, which was responsible for the 2015 epidemic in French Polynesia [40], during 24 h in the presence of increasing concentrations of *P. phillyreifolius*. At non-cytotoxic concentrations, the amount of intracellular viral RNA determined by RT-qPCR was reduced up to 3.5 log (Figure 1C). Quantification of viral growth by plaque-forming assay revealed that ZIKV-PF13 was sensitive to different *P. phillyreifolius* concentrations (Figure 1D). Indeed, as observed for the African strain ZIKV^GFP^, *P. phillyreifolius* lowered ZIKV-PF13 progeny production up to 3-log at non-cytotoxic doses with a dose-dependent manner (Figure 1D). In parallel, viral protein production was evaluated 24 h post infection (hpi) by immunofluorescence assay using an anti-flavivirus E mAb 4G2 (Figure 1E). Our data showed that the production of the viral protein E in A549 cells was severely affected after *P. phillyreifolius* treatment even at lower concentrations (Figure 1E). Together, these results showed that *P. phillyreifolius* exerts a potent antiviral effect against an African and an Asian strain of ZIKV.

**Figure 2:**
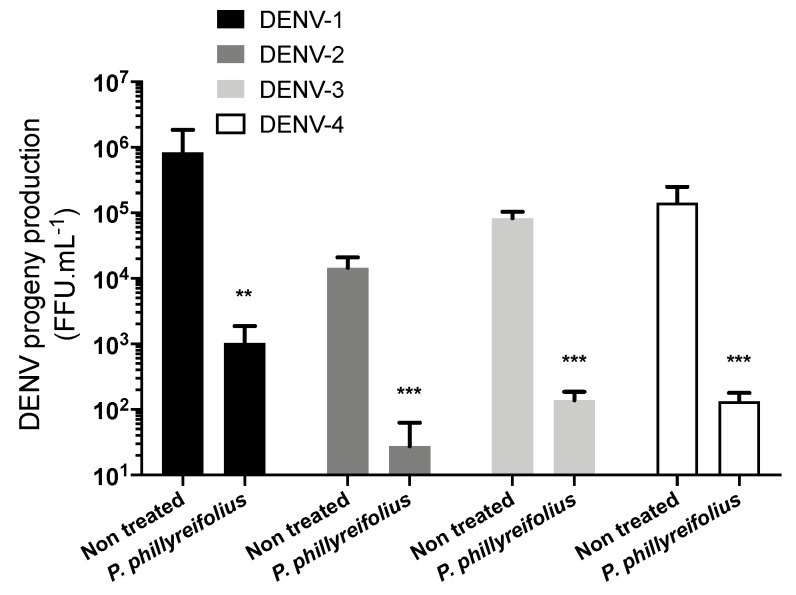
*Phyllanthus phillyreifolius* extract inhibits infection of four dengue virus (DENV) serotypes in Huh7.5 cells. Huh7.5 cells were infected for 48 h with DENV at different MOI 0.2, 2, 0.5, and 2 for DENV 1-4, respectively, with or without *P. phillyreifolius* (250 µg/mL). The virus progeny production was titrated in C6/36 cells using a foci-forming immunodetection assay. Data represent the means ± SD from three independent experiments performed in duplicate. ** *p* < 0.01; *** *p* < 0.001, n.s. = not significant.

We asked whether *P. phillyreifolius* extract also exerts antiviral activity against DENV, a closely related flavivirus to ZIKV. The antiviral activity of *P. phillyreifolius* was assessed on the four different serotypes (clinical isolates for DENV-1 to DENV-3 and a DENV-4 laboratory-adapted strain). The human hepatoma cell lines Huh7.5, which are permissive to DENV infection, were used to perform these experiments (Figure 2). Production of infectious DENV particles from Huh7.5 cells in presence of *P. phillyreifolius* (250 µg/mL) was quantified using foci forming immunodetection assays (Figure 2). A decrease of up to 3-log of DENV infectivity was observed upon DENV1-4 (Figure 2). These results show that *P. phillyreifolius* extract exhibits potent antiviral activity against the four DENV serotypes.

### 2.2. Geraniin, the Major Compound of Phyllanthus phillyreifolius Ethanolic Extract, Inhibits ZIKV Infection at Non-Cytotoxic Concentrations

We recently characterized the phytochemical composition of *P. phillyreifolius* ethanolic extract using UPLC-QTOF-MS [32]. We have demonstrated that *P. phillyreifolius* extract contains six phenolic components in which geraniin is the major quantitatively constituent, followed by ellagic acid, elaeocarpusin, rutin, quercetin, and gallic acid [32,33]. It has been reported that geraniin is a potent inhibitor of DENV-2 [34]. Consequently, DENV served as a flavivirus positive control in the following experiments.

**Figure 3:**
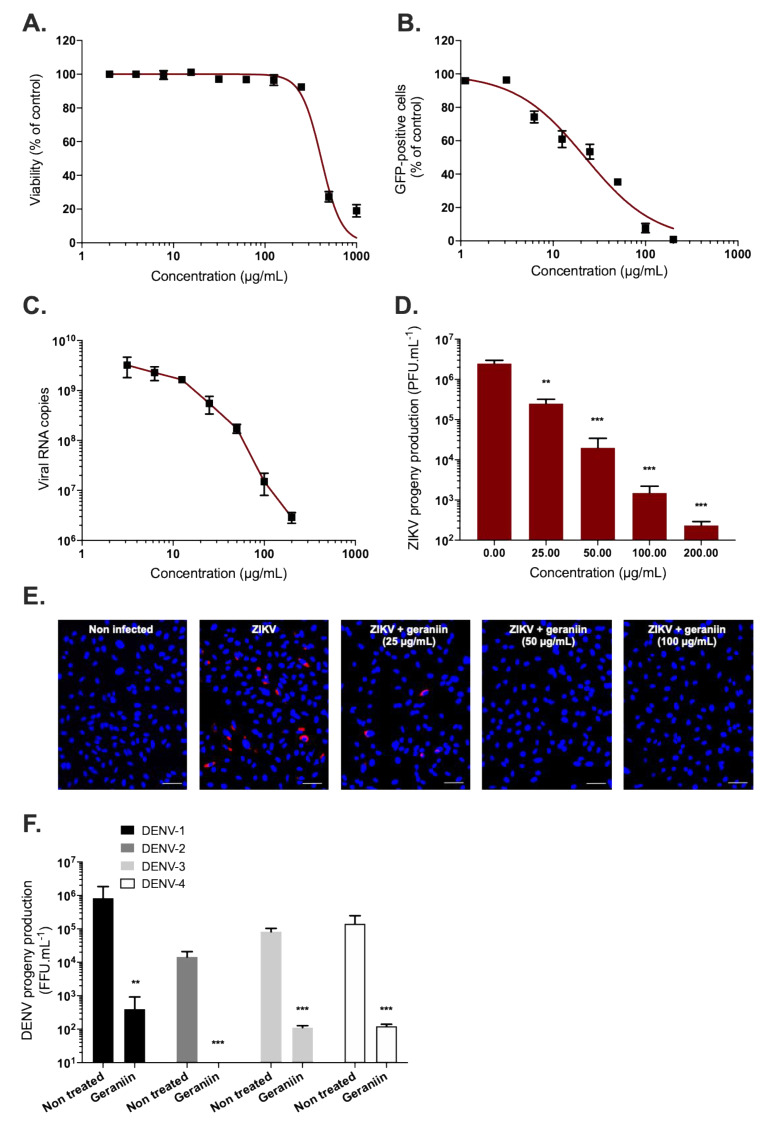
Geraniin inhibits ZIKV infection at non-cytotoxic concentrations. (**A**) A549 cells were incubated with two-fold serial dilutions (1000 to 2 µg/mL) of geraniin for 72 h. Cell viability was evaluated using an MTT assay. (**B**) A549 cells were infected with ZIKV^GFP^ at MOI of 1 in presence of different concentrations (200, 100, 50, 25, 12.5, 6.25, 3.125, and 1.125 µg/mL) of geraniin. Flow cytometric analysis of GFP fluorescence was performed 24 h post infection. A549 cells were infected with ZIKV-PF13 at MOI of 2 and continuously incubated with increasing concentrations of geraniin. (**C**) The amount of viral genomic RNA in ZIKV-PF13-infected A549 cells was determined by RT-qPCR. (**D**) ZIKV progeny production was quantified by plaque-forming assay in presence of different concentrations of geraniin (200, 100, 50, and 25 µg/mL). The results shown are means ± SD of four independent experiments and are expressed as relative value compared to untreated infected cells. ** *p* < 0.01; *** *p* < 0.001, n.s. = not significant (**E**) Immunofluorescence analysis of viral protein expression in ZIKV-PF13-infected A549 cells. The ZIKV (red) and nuclei (blue) were visualized by fluorescence microscopy. Scale bars are 50 μm. Results from a representative experiment (n = 3 repeats) are shown. (**F**) DENV was used as a flavivirus positive control. Huh7.5 cells were infected for 48 h with DENV at different MOI 0.2, 2, 0.5 and 2 for DENV 1-4 respectively with or without geraniin (200 µg/mL). The virus progeny production was titrated in C6/36 cells using a foci-forming immunodetection assay. Data represent the means ± SD from three independent experiments performed in duplicate.

Thus, we decided to investigate the anti-ZIKV activity of geraniin. We first tested its cytotoxicity on A549 cells using an MTT assay (Figure 3A). The CC_50_ was calculated as 420 µg/mL (Table 1). The concentration of 200 µg/mL of geraniin, which maintained 95%–100% of A549 cell viability (Figure 3A), was chosen to test the potential antiviral activity of geraniin on A549 cells. As previously described for *P. phillyreifolius*, the antiviral activity of geraniin was assessed using ZIKV^GFP^. FACS assay showed that geraniin severely restricted ZIKV infection in A549 cells, yielding 90% inhibition of infection at 100 µg/mL (Figure 3B). The IC_50_ value was 22 µg/mL (Table 1). Based on the determined cytotoxicity and antiviral efficacy, the Selectivity Index (SI) was estimated, according to the values of CC_50_ and IC_50_ (SI = CC_50_/IC_50_), as 13 and 19 for *P. phillyreifolius* and geraniin, respectively (Table 1).

The antiviral activity of geraniin was next evaluated against the Asian epidemic ZIKV strain (ZIKV-PF13). For this purpose, A549 cells were inoculated with ZIKV-PF13 in presence of non-cytotoxic concentrations of geraniin. Dose–response RT-qPCR analysis showed that geraniin severely restricted ZIKV-PF13 replication in A549 cells yielding a 3-log reduction of viral RNA copies at 200 µg/mL (Figure 3C). Likewise, ZIKV-PF13 progeny production was severely inhibited in a concentration-dependent manner using a plaque forming assay (Figure 3D). Geraniin reduced the viral progeny production by at least 3.5-log at 200 µg/mL (Figure 3D). In parallel, immunofluorescence analysis using anti-flavivirus E mAB 4G2 confirmed that geraniin severely restricted E-ZIKV production in A549 cells 24 hpi (Figure 3E). DENV1-4 were used as a flavivirus positive control to assess the antiviral activity of geraniin (Figure 3F). As expected from a previous study [34], geraniin severely restricted DENV-2 growth, as well as growth of the three other DENV serotypes, in Huh7.5 at non-cytotoxic concentrations. Indeed, as observed for ZIKV, geraniin lowered DENV progeny production of the four DENV serotypes up to 3-log at 200 µg/mL. Altogether, these data demonstrate that geraniin can efficiently inhibit ZIKV and DENV infection in human cells in a dose-dependent manner and reflect its potential as a source of natural antiviral phytochemical.

### 2.3. Phyllanthus phillyreifolius Ethanolic Extract and Geraniin Prevent ZIKV Entry in A549 Cells

**Figure 4:**
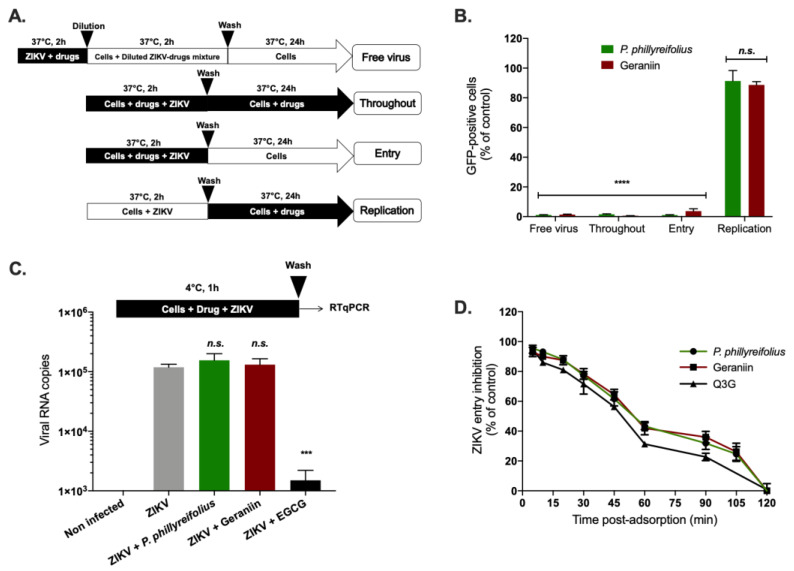
*Phyllanthus phillyreifolius* extract and its major component geraniin target the early stage of ZIKV infection. (**A**) Schematic representation of time-of-drug addition assays used to characterize the antiviral activity of *P. phillyreifolius* extract (250 µg/mL) and geraniin (200 µg/mL). (**B**) flow cytometric analysis of GFP expression in A549 cells infected with ZIKV^GFP^ during 24 h at MOI of 1 under the different experimental conditions shown in (**A**). *** *p* < 0.001, n.s. = not significant. (**C**) A549 cells were infected with ZIKV at MOI of 1 for 1 h at 4 °C with or without *P. phillyreifolius* (250 µg/mL) or geraniin (200 µg/mL). Epigalocathechin gallate (EGCG) (100 µM) was used as positive control. The number of virus particles bound to cell surface was measured by RT-qPCR. (**D**) A549 cells were incubated 1 h with ZIKV^GFP^ at 4 °C and the temperature was shifted to 37 °C in absence (vehicle) or presence of 250 µg/mL of *P. phillyreifolius* or 200 µg/mL of geraniin which have been added at different time points post temperature shift. Q3G (200 µg/mL) was used as a positive control. ZIKV entry inhibition was calculated from the percentage of GFP-positive cells determined at 24 hpi. Data represent the means ± SD from four independent experiments performed in triplicate.

Time-of-drug addition approach [41] was performed to determine which stages of ZIKV infection are targeted by *P. phillyreifolius* and whether geraniin is the main active antiviral component present in plant extract (Figure 4A). When A549 cells were treated with a non-cytotoxic concentration of geraniin (200 µg/mL) or *P. phillyreifolius* (250 µg/mL) throughout the experiment or concomitantly with virus input for 2 h (entry), the fluorescence intensity representing viral replication was detected in less than 5% of cells (Figure 4B, “Throughout”, “Entry”). However, no significant effect was observed when *P. phillyreifolius* or geraniin were added 2 h post adsorption (Figure 4B, “Replication”). These results suggest that the antiviral activity of *P. phillyreifolius* and geraniin was not mediated by the inhibition of the replication stage but rather by the inhibition of early steps of viral infectious cycle. To investigate whether *P. phillyreifolius* extract or geraniin render virus particles unable to initiate viral infection in the host cell (Figure 4A, “Free virus”), ZIKV^GFP^ particles were incubated 2 h at 37 °C with *P. phillyreifolius* (250 µg/mL) or geraniin (200 µg/mL) then diluted 50-fold prior to A549 cell infection. This dilution titrates the plant extract or geraniin below their therapeutic concentrations and prevents potential interactions with the host cell surface. Our results showed that such treatment of ZIKV^GFP^ resulted in 99% reduction of GFP-positive cells at 24 hpi compared to cells infected with untreated ZIKV^GFP^ (Figure 4B, Free virus). These results suggest that phytochemicals present in *P. phillyreifolius* extract, mainly geraniin, interact with ZIKV-free particles, irreversibly, to prevent infection. This observation suggests that geraniin could bind to ZIKV particles resulting in disassembly of ZIKV particles or affecting their infectivity. To investigate this hypothesis, an RNase protection assay [25] was performed to determine whether *P. phillyreifolius* extract causes virus particle disassembly leading to a release of genomic RNA (Figure S1). Given that viral genomic RNA displayed a complete resistance to RNase A (Figure S1), we can exclude the hypothesis that *P. phillyreifolius*-mediated inhibition of ZIKV relates to a loss of virion integrity. Taken together, these results suggest that *P. phillyreifolius* extract acts in preventing the initiation of the virus infectious cycle, presumably through a blockade of the virus entry steps into the host cell.

To determine whether geraniin and/or other phytochemicals present in *P. phillyreifolius* extract preclude the attachment of ZIKV particles to cell membrane, prechilled ZIKV^GFP^ and *P. phillyreifolius* or geraniin were mixed and allowed to bind A549 cells at 4 °C for 1 h to allow virus binding and prevent its entry (Figure 4C). Epigalocathechin gallate (EGCG), which is known to inhibit ZIKV attachment was used as a positive control [29,41]. After 1 h of ZIKV attachment in presence or absence of *P. phillyreifolius* or geraniin, cells were rinsed and the number of attached ZIKV particles was evaluated using RT-qPCR (Figure 4C). Our data showed that the number of viral particles attached to the cell surface in presence of *P. phillyreifolius* or geraniin was similar to untreated cells (Figure 4C). As expected from previous studies, the amount of ZIKV particles attached to cell membrane was severely reduced in the presence of EGCG [29,41]. These results suggest that neither geraniin nor any other phytochemical present in plant extract were capable of interfering with the ZIKV attachment step.

The effect of *P. phillyreifolius* extract and its main compound on a subsequent post-adsorption step of the virus infectious cycle was next analyzed, thereby testing their effect on the kinetics of ZIKV internalization (Figure 4D). Thus, ZIKV^GFP^ was allowed to bind to the cell surface at 4 °C followed by a shift of temperature to 37 °C. At different time points after temperature shift, cells were washed and then incubated with *P. phillyreifolius* extract or geraniin (Figure 4D). ZIKV-infected A549 cells were examined for GFP-expression at 24 hpi. The natural flavonoid isoquercitrin (Q3G), which is known to inhibit ZIKV internalization, was used as positive control [25]. Flow cytometric analysis showed that *P. phillyreifolius* extract and geraniin are capable of inhibiting internalization step of ZIKV in a similar way to that of Q3G (Figure 4D). Until 15 min after temperature shift, geraniin treatment resulted in a complete inhibition of viral entry with a reduction of GFP-positive cells by 90% (Figure 4D). Neither *P. phillyreifolius* extract nor geraniin are able to inhibit viral infection when added 2 h post temperature shift. Moreover, both *P. phillyreifolius* extract and geraniin have an identical internalization inhibition profile, thus suggesting that geraniin is probably the main active compound present in the extract of *P. phillyreifolius*. These kinetics are also similar to those of the positive control Q3G (Figure 4D). These results suggest that geraniin-mediated inhibition of ZIKV infection occurs early after virus binding to the cell membrane and could be explained by the incapacity of the ZIKV-attached particles to be internalized into the host cell in presence of geraniin.

### 2.4. Phyllanthus phillyreifolius and Geraniin Do Not Exhibit Acute Toxicity in Zebrafish

Zebrafish is a model organism widely used for studying in vivo toxicity during development and adulthood. It represents an interesting alternative to toxicity studies in rodents. In addition to sharing a high genomic homology with humans (>70%) [42], zebrafish also display many physiological processes common with mammals [43] that are interesting for toxicity studies, such as liver and renal metabolic functions. Consequently, the potential toxicity of *P. phillyreifolius* and geraniin was assessed in adult zebrafish as recently done for other medicinal plant extract such as *Ayapana triplinervis* essential oil [44]. To this aim, intraperitoneal injection was performed with the corresponding maximum non-toxic concentration (MNTC) of 375 microg/g and 300 microg/g of body weight for *P. phillyreifolius* and geraniin, respectively (Table 2). Any striking signs of stress, suffering, feeding behavior, and abnormal behavior were carefully checked several times a day for a period of 5 days. As shown in Table 2, the injection of *P. phillyreifolius* and geraniin did not impact fish behavior (feeding and locomotion), and no sign of stress was reported (Table 2). Moreover, the *P. phillyreifolius* and geraniin treatments resulted in 100% fish survival, similar to the control injection with PBS (Table 2). Taken together, these data suggest that these doses do not exhibit acute toxic effects.

### 2.5. Geraniin Does Not Disturb Zebrafish Locomotor Activity

**Figure 5:**
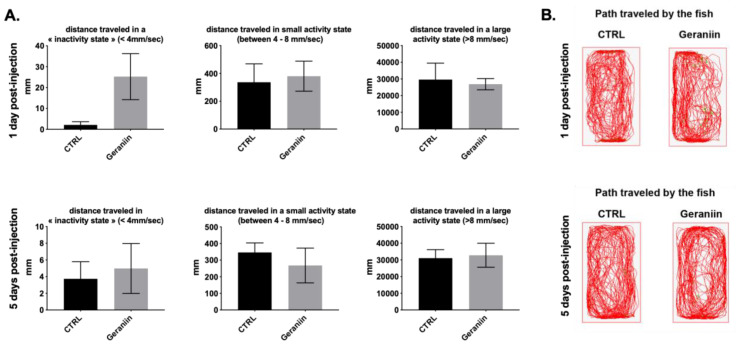
Geraniin did not impair fine-tuned locomotor activity at 1 day and 5 days post injection. (**A**) distance travelled in “inactivity” (<4 mm/s), small activity (4–8 mm/s), and large activity (>8 mm/s) states in fish injected with geraniin at 1 day post injection and 5 days post injection. Note that no significant differences were observed compared with the PBS-injected fish (CTRL). (**B**) representative paths traveled by PBS-injected fish and geraniin-injected fish at 1 and 5 days post injection. Data represent the means ± SEM.

In order to have a better idea of the potential effects of geraniin on the fine-tuned locomotor behavior, as a sign of stress, the locomotor activity of zebrafish was carefully monitored by performing a ZebraCube analysis (Figure 5). After a 10 min period of adaptation into their new tanks, the locomotor activity of individual vehicle and geraniin-injected fish was monitored for a 10 min period at 1 and 5 days post injection (dpi). The results demonstrate that geraniin-injected fish traveled the same distance in an “inactive” state and in small and large activity states as their respective controls at 1 dpi and 5 dpi (Figure 5A). In addition, the path traveled by vehicle and geraniin-injected fish are largely similar establishing that the geraniin injection has no obvious effect on the fine-tuned locomotor activity of fish (Figure 5B). Consequently, it appears that the MNTC injection of geraniin did not induce adverse effects in zebrafish.

## 3. Discussion

In the absence of effective antivirals against ZIKV and DENV, these arthropod-borne flaviviruses have emerged as a serious health concern that affected millions of people in the last years [2,45]. Albeit a huge amount of research having been conducted in the last five years, there is still a lack of effective anti-ZIKV compounds. Within a project aiming to investigate the antiviral activity of Reunion Island (RUN) plant biodiversity, we recently identified several endemic or indigenous polyphenol-rich medicinal plants from RUN that inhibit ZIKV infection by preventing viral entry [41,46,47]. As we have recently demonstrated the strong antioxidant activity of *P. phillyreifolius* due to its high polyphenol content, mainly ellagitannin such as geraniin [32,33], we decided to investigate the anti-ZIKV activity of *P. phillyreifolius* and its major compound geraniin. We demonstrated here, using RT-qPCR, immunofluorescence, and plaque forming assays, that both African and Asian epidemic ZIKV strains [6] are sensitive to cell treatment with a non-cytotoxic concentrations of *P. phillyreifolius* and its major compound geraniin. We also demonstrated that *P. phillyreifolius* extract prevents infection of human cells by four DENV serotypes. We showed that *P. phillyreifolius* extract acts, probably through its major compound geraniin, on early steps of the viral replication cycle, in a similar way than isoquercitrin (Q3G), a natural flavonoid, which is known to inhibit ZIKV internalization into the host cell [25]. Indeed, RNase protection, inactivation, binding, and internalization assays suggest that *P. phillyreifolius*-mediated inhibition of ZIKV was not associated with a loss of viral integrity but rather with a neutralization of ZIKV infectivity allowing attachment and preventing internalization of ZIKV particles in the host cell. Geraniin-mediated inhibition of ZIKV is different from that described previously where geraniin extracted from *Nephelium lappaceum* inhibits DENV-2 binding to the host cell [34].

*Phyllanthus* is one of the largest genera in the family *Phyllanthaceae* that comprises over 700 species distributed mainly in the tropics and subtropics and is in constant use in traditional medicine to treat diverse human diseases [48]. *Phyllanthus* species represent an extensive phytochemical diversity, e.g., tannins, terpenes, alkaloids, flavones, saponins, etc. These chemical compounds derived from *Phyllanthus* species exhibit antiviral effects against a broad spectrum of enveloped RNA and DNA viruses [49,50,51,52,53,54,55]. The results presented in this study underscore endemic medicinal plants from the Mascarene islands as a promising source of antivirals and add *Phyllanthus phillyreifolius* to the group of *Phyllanthus* species exhibiting antiviral effects against medically relevant flavivirus.

Importantly, in addition to this antiviral activity, we demonstrated that *P. phillyreifolius* extract, as well as its major compound geraniin, do not show acute toxicity in vivo using a zebrafish model, relevant for drug toxicity assessment [43,44,56,57,58]. Our zebrafish assay showed that neither the plant extract nor its major compound geraniin impact fish behavior (feeding and locomotion), and no sign of stress was reported during the 5 days of investigation following drug exposure. The absence of striking behavioral effects at the effective antiviral concentration of geraniin on fish behavior (feeding, swimming, locomotor activity, stress), suggests that this concentration should be safe for further pre-clinical studies. These innocuity and non-deleterious effect at effective antiviral doses further indicate that the medicinal plant *P. phillyreifolius* is a potential source for the development of natural and safe antiviral drugs to fight ZIKV and DENV infections.

## 4. Materials and Methods

### 4.1. Cells, Viruses and Reagents

Human lung epithelial A549 cells (ATCC, CCL-185, Manassas, VA, USA) and Vero cells (ATCC, CCL-81) were grown in Eagle minimum essential medium (MEM: Gibco/Invitrogen, Carlsbad, CA, USA) supplemented with 10% and 5%, respectively, of heat-inactivated fetal bovine serum (FBS Good: Invitrogen), 2 mmol.L^−1^ L-Glutamine, 1 mmol.L^−1^ sodium pyruvate, 100 U/mL of penicillin, 0.1 mg/mL of streptomycin, and 0.5 µg/mL of Amphotericin B (PAN Biotech, Aidenbach, Germany) under a 5% CO_2_ atmosphere at 37 °C. The recombinant Zika virus molecular clone expressing the GFP reporter gene (ZIKV^GFP^) and the clinical isolate PF-25013-18 of ZIKV (ZIKV-PF13) have been previously reported [39,40]. DENV1-4 have been previously reported [47]. DENV stocks were grown in C6/36 cells and titrated by foci-forming immunodetection assay. EGCG, Q3G, and geraniin were purchased from Sigma-Aldrich. The growth culture medium supplemented with 0.2% of Dimethyl sulfoxide (DMSO) was used as a vehicle control.

### 4.2. Extraction and Phytochemical Characterization of P. phillyreifolius Extract

The leaves of *P. phillyreifolius* were collected in the southwest of Reunion Island in November 2013 and immediately subjected to drying overnight at 37 °C. 5 g of dried and milled (to 1 mm particle size) plant material was extracted twice by stirring with 100 mL ethanol:water (70:30, *v*/*v*) at room temperature for 1 h, the extract filtered over Whatman No.1 filter paper and combined. We selected a traditional extraction method, which has been widely used for medicinal herbs. The method was proven to guarantee the effective and safe recovery of sensitive bioactive molecules. After extraction, ethanol was removed in a rotary evaporator (Büchi, Flawil, Switzerland), while residual water was freeze dried. The yield of obtained extract was 33.63 ± 1.15%. Dry extract was stored in a freezer prior to analysis.

The component analysis of extract was performed on an UPLC system equipped with a photodiode array (PDA) detector (Waters, Milford, MA, USA) according to the procedure previously described by [32]. The separation was carried out on Acquity BEH, C18 column (100 mm × 2.1 mm, 1.7 µm) at a column temperature of 40 °C. The mobile phase consisted of solvents 0.1% formic acid in ultrapure water (A) and 100% acetonitrile (B) with a linear gradient programmed as follows: 0.0–14 min, 5% B; 15–17 min, 100% B; 18 min, 5% B. The flow rate was 0.4 mL/min and the injection volume 1 µL. The UPLC system was coupled to quadrupole-time of flight mass spectrometer (Q-TOF) equipped with an electrospray ionization probe and controlled by HyStar software (Bruker Daltonic, Bremen, Germany). MS data were recorded in ESI negative ionization mode over a mass range from *m*/*z* 80 to 1200, the capillary voltage was maintained at +4000 V. The peaks were identified by comparing their retention times and parent ions with external standards, references, and commercial databases.

The quantification of geraniin content was performed using Acquity UPLCTM H-Class equipped with Xevo TQ-S tandem quadrupole mass spectrometer (Waters, Milford, MA) as reported elsewhere [32]. Chromatographic separation was performed using the same column and solvents as described above with a linear gradient programmed as: 0.0–7 min, 5% B; 8–9 min, 50,7% B; 10–11 min, 100% B; 12–20 min, 5% B. The flow rate was 0.4 mL/min, and the sample injection volume was 5 µL. MS detection was achieved in the single-ion-monitoring (SIM) mode. The *m*/*z* values and dwell times of geraniin were set as 951.1957 *m*/*z* at 0.050 s. The concentration of geraniin in extract was calculated from calibration curve prepared using geraniin standard at concentrations of 0.05–50 µg/mL (y = 6158x + 4127; R^2^ = 0.9986). Geraniin concentration in extract was 327 mg/g DWE. To evaluate the recovery of geraniin from 1 g of dried plant, this concentration was recalculated to g of dry weight of plant (DWP) and resulted 110 mg/g DWP.

### 4.3. MTT Assay

Two-fold dilutions of plant extract and geraniin ranging from 1000 µg/mL to 2 µg/mL were used to treat A549 cells at a density of 1.5 × 10^4^ cells per well cultured in 96-well culture plates. Cells were rinsed with PBS 1X and 120 µL of culture medium mixed with 5 mg/mL MTT (3-[4,5-dimethylthiazol-2-yl]-2,5- diphenyltetrazolium bromide) solution was added 24 h post incubation at 37 °C. The cells were re-incubated for 2 h at 37 °C. MTT medium was then removed, and the formazan crystals were solubilized with 50 µL of DMSO. Absorbance was measured at 570 nm with a background subtraction at 690 nm. The CC_50_ was determined using a nonlinear regression on Graphpad prism software.

### 4.4. Flow Cytometry Assay

Cells were rinsed twice with PBS1X, trypsinated with 30 µL of trypsin/EDTA, and then fixed with 3.7% PFA in MEM for 15 min. The cells were then subjected to a flow cytometric analysis using Cytoflex (Beckman Coulter, Brea, CA, USA). Results were analyzed using cytExpert software (Brea, CA, USA).

### 4.5. RT-qPCR

Total RNA including genomic viral RNA was extracted from cells with RNeasy kit (Qiagen, Hilden, Germany) and reverse transcribed using E reverse primer (5’-TTCACCTTGTGTTGGGC-3’) and M-MLV reverse transcriptase (Life Technologies, Villebon-sur-Yvette, France) at 42 °C for 50 min. Quantitative PCR was performed on a CFX96 Real-Time PCR Detection System (Applied Biosystems, Life Technologies, Villebon-sur-Yvette, France). Viral genomic cDNA was amplified using 0.2 μM of each primer and GoTaq Master Mix (Promega, Charbonnières-les-bains, France). For each single-well amplification reaction, a threshold cycle (Ct) was calculated using the CFX96 program (Bio-Rad) in the exponential phase of amplification. A synthetic gene coding for nucleotides 954 to 1306 of the MR766 strain (GenBank: LC002520) cloned in the pUC57 plasmid was used as template to generate a standard curve, which then served to make an absolute quantitation of bound viruses.

### 4.6. Plaque Forming Assay

Plaque forming unit assay was used to quantify virus infectious particles. Vero cells were seeded the previous day in 24-well culture plates at a density of 7 × 10^4^ cells per well. Cells were infected by 0.15 mL of tenfold dilutions of supernatant. After 2 h of incubation at 37 °C, 0.2 mL of culture medium supplemented with 5% fetal bovine serum (FBS) and 0.8% carboxymethylcellulose sodium salt (Sigma-Aldrich) was added, and the incubation was extended for 96 h at 37 °C. Cells were rinsed with PBS 1X, fixed with PFA 3.7 % in PBS 1X, and stained with 0.5% crystal violet (Sigma-Aldrich, Saint-Quentin-Fallavier, France) diluted in 20% ethanol. Plaques were counted and expressed as plaque-forming unit per mL (PFU/mL)

### 4.7. Immunofluorescence Assay

Cells were rinsed twice with PBS1X, fixed with PFA, 3.7% in PBS1X, and permeabilized for 5 min (PBS 1X 0.15% Triton X-100). Cells were then stained 1h at room temperature in the dark for ZIKV E protein using 4G2-Alexa 594 (1:1000 in PBS-BSA 2%). DAPI staining was used to label cells nuclei. Coverslips were mounted in Vectashield, and the fluorescence was observed using Nikon Eclipse E2000-U microscope. Hamamatsu ORCA-ER camera and NIS-Element AR (Nikon) imaging software were used to capture images.

### 4.8. Foci-Forming Immunodetection Assay

C6/36 cells were seeded at a density of 1 × 10^5^ in 24-well plates and incubated overnight at 37 °C. Tenfold serial dilutions of supernatant were prepared in duplicate in culture medium and 0.4 mL of each dilution was added to cells for 90 min. Then the inoculum was removed and a CMC media (L-15 supplemented with 10% FBS, 0.52% tryptose, 50 mg/mL gentamicin, 1.6% carboxymethylcellulose) was added. After seven days of incubation, immunostaining was performed using the mouse monoclonal antibody 4G2 followed by goat-antimouse immunoglobulin conjugated to alkaline phosphatase (Promega, Madison, WI, USA). A solution of NBT (nitroblue tetrazolium chloride) and BCIP (5-bromo-4-chloro-39-indolyphosphate p-toluidine salt) (Promega, Madison, WI, USA) was used as substrate to detected antibodies. Foci were counted and expressed as FFU/mL.

### 4.9. Virus Binding Assay

A549 cells were seeded in 12-well plates at a density of 2 × 10^5^ cell per well. Cells were incubated with ZIKV at MOI of 1 in the presence or absence of plant extract or geraniin for 1 h at 4 °C. The inoculum was then removed, and cells were washed with cold MEM supplemented with 2% FBS. Samples were then submitted to RT-qPCR.

### 4.10. Internalization Assay

A549 cells were seeded in 24-well plates at a density of 1.5 × 10^5^ cells per well. Cells were incubated with ZIKV^GFP^ MOI of 1 for 1 h at 4 °C. Cells were then shifted to 37 °C in the presence or absence of plant extract or geraniin. The latter have been added at different time points, post temperature shift, covering the entry step of ZIKV infection. Cells were further incubated until 24 h at 37 °C. Cells were fixed with PFA 3.7% and then submitted to flow cytometry assay.

### 4.11. Virus Inactivation Assay

ZIKV^GFP^ free particles (5 × 10^5^ PFU) were incubated with the plant extract or geraniin for 2 h at 37 °C. As control, ZIKV^GFP^ was incubated with MEM supplemented with 0.2% of DMSO. The mixture was diluted to 40-fold prior to infection and then inoculated on A549 cells. The supernatant was discarded after 2 h of incubation at 37 °C. Cells were washed twice with PBS1X and then incubated for 22 h at 37 °C before being subjected to cytometry assay.

### 4.12. Fish Maintenance, Intraperitoneal Injection, and Locomotor Activity Recording

Adult wildtype zebrafish (Danio rerio; 3–6 months male) were maintained under standard conditions of temperature (28 °C), photoperiod (14/10 h light/dark), and conductivity (400 μS). Every day, fish were fed 3 times with commercially available food from Planktovie (Gemma Micro ZF 300). For intraperitoneal injections, fish were anesthetized with 0.02% tricaine (MS-222; REF: A5040, Sigma-Aldrich) and injected with the respective vehicle (1X PBS, CTRL), *Phyllanthus phillyreifolius* extract (375 microg.g^−1^ of body weight) and its main active compound Geraniin (300 microg.g^−1^ of body weight). After injection, fish were immediately placed back in fish water and carefully observed for detecting any striking signs of stress, locomotor activity, and feeding behavior issues. At 1 and 5 days post injection (dpi), 3 CTRL and geraniin-injected fish were monitored for locomotor activity using the ZebraCube equipment (Viewpoint). Individual fish were placed in separate tanks containing 250 mL of water within the ZebraCube. A separation was placed between the CTRL and geraniin tanks in order to avoid visual interaction between the fish from the two groups. Before monitoring the locomotion, fish were adapted to their new tank for 10 min. Then, the locomotor activity was recorded for 10 min defining inactivity (<4 mm/s), small activity (4–8 mm/s), or high activity (>8 mm/s) states. A total number of 3 CTRL fish versus 3 geraniin fish were subjected to behavioral analysis at 1 dpi, and 5 CTRL fish versus 5 geraniin fish at 5 dpi. All animal experiments were conducted in accordance with the French and European Community Guidelines for the Use of Animals in Research (86/609/EEC and 2010/63/EU) and approved by the local Ethics Committee for animal experimentation of CYROI (APAFIS #2019052910002738_v4). At the end of the procedure, the animals were sacrificed with an overdose of tricaine.

### 4.13. Data Analysis

Comparison between different concentrations was done by a one-way ANOVA test. All values were expressed as mean ± SD of at least three independent experiments. All statistical tests were done using the software Graph-Pad Prism (version 8.0; La Jola, CA, USA). Values of *p* < 0.05 were considered statistically significant for a Dunnett’s multiple comparisons test. Degrees of significance are indicated on the figure as follow: ** *p* < 0.01; *** *p* < 0.001, n.s. = not significant.

## Figures and Tables

**Table 1:** Cytotoxicity and antiviral activity of *P. phillyreifolius* and geraniin against ZIKV.

Compound	CC_50_ (µg/mL) ^a^	IC_50_ (µg/mL) ^b^	SI ^c^
*P. phillyreifolius*	715 ± 18	55 ± 2.3	13
Geraniin	420 ± 16	22 ± 6.8	19

Cytotoxic concentration (CC_50_) and inhibitory concentration (IC_50_) were obtained by performing nonlinear regression followed by the construction of a sigmoidal concentration–response curves from Figure 1A,B. ^a^ Concentration that inhibited cell viability by 50%; ^b^ concentration that inhibited infection by 50%; ^c^ selectivity index (CC_50_/IC_50_).

**Table 2:** Survival and behavior of fish injected with *Phyllanthus phillyreifolius* extract and geraniin from 1 day post injection to 5 days post injection (dpi).

		Number of Fish Alive					
	Number of Injected Fish	1 dpi	2 dpi	3 dpi	4 dpi	5 dpi	Survival Rate at 5 dpi (%)
1x PBS (vehicle)	7	7	7	7	7	7	100
*P. phillyreifolius*	8	8	8	8	8	8	100
Geraniin	8	8	8	8	8	8	100
Feeding behavior		normal	normal	normal	normal	normal	
Locomotor behavior		normal	normal	normal	normal	normal	

## Supplementary Materials

The following are available online, Figure S1: Effect of *P. phillyreifolius* on ZIKV infectivity.
