# Supplementary material for: The Geraniin-Rich Extract from Reunion Island Endemic Medicinal Plant Phyllanthus phillyreifolius Inhibits Zika and Dengue Virus Infection at Non-Toxic Effect Doses in Zebrafish
Source: Molecules. 2020 May 15;25(10):2316. doi: 10.3390/molecules25102316 (PMC7287739; doi:10.3390/molecules25102316)
Supplement: Supplementary file 1 [file molecules-25-02316-s001.pdf]

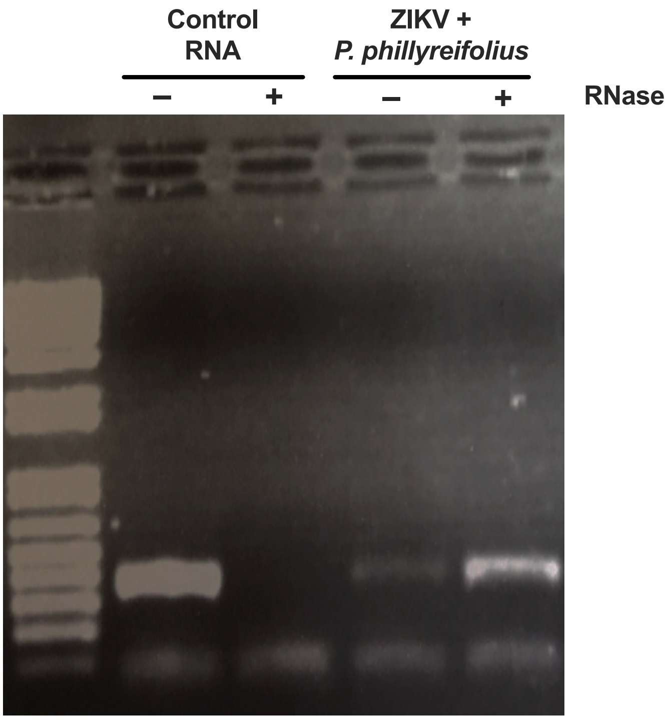

Figure S1. Effect of *P. phillyreifolius* on ZIKV infectivity. RNase A protection assay on ZIKV. ZIKV particles was incubated with 250 µg/mL of *P. phillyreifolius* extract or mock-treated in presence of RNase A for 1 h at 37°C. Viral RNA was extracted and amplified by RT-PCR with ZIKV E primers. Viral RNA extracted from ZIKV and treated with RNase A or vehicle served as controls. Results from a representative experiment (n = 3 repeats) are shown.
